# Intestinal Epithelial Toll-Like Receptor 4 Signaling Affects Epithelial Function and Colonic Microbiota and Promotes a Risk for Transmissible Colitis

**DOI:** 10.1128/IAI.01374-15

**Published:** 2016-02-24

**Authors:** Rishu Dheer, Rebeca Santaolalla, Julie M. Davies, Jessica K. Lang, Matthew C. Phillips, Cristhine Pastorini, Maria T. Vazquez-Pertejo, Maria T. Abreu

**Affiliations:** a Division of Gastroenterology, Department of Medicine, University of Miami, Miller School of Medicine, Miami, Florida, USA; b Department of Microbiology and Immunology, University of Miami, Miller School of Medicine, Miami, Florida, USA; c Department of Pathology, Palms West Hospital, Loxahatchee, Florida, USA

## Abstract

Evidence obtained from gene knockout studies supports the role of Toll-like receptor 4 (TLR4) in intestinal inflammation and microbiota recognition. Increased epithelial TLR4 expression is observed in patients with inflammatory bowel disease. However, little is known of the effect of increased TLR4 signaling on intestinal homeostasis. Here, we examined the effect of increased TLR4 signaling on epithelial function and microbiota by using transgenic villin-TLR4 mice that overexpress TLR4 in the intestinal epithelium. Our results revealed that villin-TLR4 mice are characterized by increases in the density of mucosa-associated bacteria and bacterial translocation. Furthermore, increased epithelial TLR4 signaling was associated with an impaired epithelial barrier, altered expression of antimicrobial peptide genes, and altered epithelial cell differentiation. The composition of the colonic luminal and mucosa-associated microbiota differed between villin-TLR4 and wild-type (WT) littermates. Interestingly, WT mice cohoused with villin-TLR4 mice displayed greater susceptibility to acute colitis than singly housed WT mice did. The results of this study suggest that epithelial TLR4 expression shapes the microbiota and affects the functional properties of the epithelium. The changes in the microbiota induced by increased epithelial TLR4 signaling are transmissible and exacerbate dextran sodium sulfate-induced colitis. Together, our findings imply that host innate immune signaling can modulate intestinal bacteria and ultimately the host's susceptibility to colitis.

## INTRODUCTION

Trillions of microbes coexist with mammalian cells in the gastrointestinal tract of the host in a relatively mutualistic environment (1, 2). The intestinal epithelium and its overlying mucus layer provide the physical barrier that separates the commensal microbiota from the host (3). Pattern recognition receptors, including the Toll-like receptors (TLRs), expressed by epithelial cells (ECs) recognize microbe-associated molecular patterns of the commensal bacteria and regulate the cross talk between intestinal microbes and their host (4, 5). Defects in TLR signaling and an aberrant immune response to perturbed endogenous microbiota are a few of the major factors that contribute to the perpetuation of inflammation and tissue injury in patients with inflammatory bowel disease (IBD) (6, 7).

TLR4 recognizes lipopolysaccharide (LPS) in the cell wall of Gram-negative bacteria. Studies have shown that TLR4 expression varies by the region of the intestine and is determined largely by the bacterial composition of that region (8). Conversely, TLR4 may play an important role in maintaining the fine balance between tolerogenic and inflammatory properties of gut microbiota by regulating innate immunity (9, 10). Although several studies have demonstrated that the microbiota composition of the host is influenced by the status of TLRs and their adapter proteins (11–13), others have reported no such effect (14, 15). In the context of TLR4 knockout mice, two separate studies have reported that genetics, maternal transmission, and housing conditions, rather than the absence of TLR4, have marked effects on the stool microbiota of these mice (14, 15). However, these studies have focused on the microbiota composition of the stool rather than the mucosa-associated microbiota, which is in close proximity to the host and differs in composition from that of the luminal microbiota (16). More importantly, the relationship between epithelial innate immune signaling and the microbiota, especially in the mucosa-associated population, is not known. Thus, the role of TLRs, including TLR4, in determining the microbiota composition and associated baseline phenotypes remains ambiguous.

TLR4 is normally expressed at very low levels by various cell types of the intestine, including epithelial and lamina propria mononuclear cells (9, 17, 18). Increased expression of TLR4 is observed in epithelial and lamina propria cells of IBD patients, suggesting an important role for TLR4 signaling in inflammation (19–21). In the context of infectious enteritis, TLR4 signaling facilitates pathogen colonization and dissemination and promotes infection-induced colitis (22). Using TLR4 knockout mice, we and others have demonstrated that TLR4 signaling is required for protection against epithelial injury, inflammation, and bacterial invasion (23, 24). On the other hand, we have previously reported that mice expressing a constitutively active form of TLR4 in the intestinal epithelium (villin-TLR4 mice) exhibit increased susceptibility to chemically induced colitis (25). We have also shown activation of the NF-κB signaling pathway and increased expression of chemokines and proinflammatory genes at the baseline in the intestinal ECs (IECs) of villin-TLR4 mice (26). These findings established the double-edged sword of TLR4 function in the intestine, wherein both low and excessive TLR4 signaling can promote intestinal inflammation. We have also previously shown that mice expressing TLR4 in the colonic ECs rather than myeloid cells are more susceptible to inflammation-associated colonic neoplasia (27).

Because of the close proximity of the colonic microbiota to the intestinal epithelium and the critical role played by intestinal epithelial TLR4 in microbiota recognition and inflammation, the aim of our study was to determine the impact of intestinal epithelium-specific TLR4 signaling on microbial composition and the related host responses. Previously published studies by other research groups have shown that mice lacking specific components of the innate immune system such as Nod2, inflammasome genes, or Myd88 are more susceptible to chemically induced colitis and possess altered microbiota compared to their wild-type (WT) littermates (28–30). Furthermore, by using cohousing strategies, it has been demonstrated that the microbiota of Nod2 knockout mice is capable of transmitting colitogenic properties to WT mice (28). However, studies detailing the effect of increased innate immune signaling on the microbiota and inflammation have not been published. Thus, we hypothesized that intestinal epithelium-specific constitutive TLR4 signaling would affect microbial composition, epithelial function, and colitis susceptibility. By using villin-TLR4 mice as a model of excessive TLR4 signaling, we show that epithelial TLR4-mediated interaction between the gut microbiota and the host is essential for protection against bacterial dissemination, epithelial injury, and maintenance of epithelial barrier function. We also show that TLR4 regulates the expression of antimicrobial genes by distinct mechanisms in different parts of the intestine. To our knowledge, this is the first study to find that enhanced TLR4 signaling promotes colonic inflammation through dysbiotic microbiota.

## MATERIALS AND METHODS

### Mice.

Mice hemizygous for the villin-TLR4 transgene that constitutively expresses TLR4 in the intestinal epithelium are termed villin-TLR4 mice and were generated as described previously (26). All experiments were conducted with 7- to 9-week-old littermates generated by crossing villin-TLR4 transgenic mice with C57BL/6 WT mice and obtained from consecutive litters of the same parent pair. For cohousing and dextran sodium sulfate (DSS) exposure experiments, age- and gender-matched nonlittermate C57BL/6 and villin-TLR4 mice were housed in the same cages for the duration of the experiment. Separately housed C57BL/6 mice were kept under similar conditions but without any villin-TLR4 mice housed in their cages. All mouse experiments were performed in accordance to the institutional animal care and use committee at the University of Miami.

### Bacterial culture.

Spleens and mesenteric lymph nodes (MLNs) were removed aseptically, weighed, and homogenized with a handheld tissue homogenizer in phosphate-buffered saline (PBS). Homogenized samples were cultured on tryptic soy sheep blood agar plates and incubated under aerobic and anaerobic conditions for 24 to 48 h. Colonization was expressed as the average number of CFU on aerobic and anaerobic plates per milligram of tissue.

### FITC-dextran assay.

Intestinal epithelial integrity was determined by fluorescein isothiocyanate (FITC)-dextran assay. Mice were orally gavaged with 4-kDa FITC-dextran (Sigma-Aldrich, St. Louis, MO) at a concentration of 60 mg/100 g of body weight. The concentration of FITC-dextran in serum was determined after 4 h with a Gemini EM fluorescence microplate reader (Molecular Devices, Sunnyvale, CA) at 490/525 nm.

### IEC isolation and mRNA expression.

Sections of small intestine and colon were excised following sacrifice. The small intestine was rinsed with cold Hanks balanced salt solution (HBSS), cut longitudinally, and transferred to 10 ml of cold HBSS and gently agitated to removal additional debris. The tissue was then cut into 1-cm pieces and transferred to cold HBSS containing 3 mM EDTA for 30 min on ice with agitation at 200 rpm on an orbital shaker. Pieces were transferred to cold HBSS and shaken vigorously for 3 to 5 min. Released ECs in the supernatant were filtered through a 70-μm filter. Cells were washed twice with 1% fetal bovine serum in HBSS to remove any residual EDTA. Colons were processed similarly to small intestines but were incubated in 60 mM EDTA in HBSS for 1 h. Total RNA was extracted from IECs or colon tissues with RNA bee according to the manufacturer's instructions (Tel-test, Friendswood, TX). A total of 1 μg of RNA was reverse transcribed with Transcriptor Reverse Transcriptase enzyme and random hexamers (Roche Life Science, Indianapolis, IN). Real-time quantitative PCR (RT-qPCR) was performed with SYBR Premix *Ex Taq* (Clontech Laboratories, Mountain View, CA) and a Roche LightCycler 480 (Roche, Indianapolis, IN). For information about the primer pairs used, see the supplemental material. The relative expression levels were calculated by the ΔΔ*C_T_* method after normalization to the average of endogenous control genes for β-actin and glyceraldehyde 3-phosphate dehydrogenase (GAPDH) or with the gene for GAPDH only for DSS exposure experiments.

### Western blot analysis.

Total cell lysate from colon and small intestine ECs was obtained by using M-PER protein extraction reagent (Thermo Scientific, Waltham, MA) supplemented with protease inhibitor cocktail set III (EMD Millipore Corp., Billerica, MA). Protein lysates were loaded into precast NuPAGE 10% Bis-Tris gels (Novex by Life Technologies, Grand Island, NY), electrophoresed (200 V, 1 h), and then transferred (30 V, 1.25 h) onto polyvinylidene fluoride microporous membranes. Membranes were blocked for 1 h in 5% bovine serum albumin (Sigma-Aldrich, St. Louis, MO) and incubated with the respective antibody (1:500 dilution) overnight at 4°C. Rabbit anti-occludin and rabbit anti-claudin-3 antibodies were obtained from Life Technologies (Grand Island, NY). Membranes were washed and incubated with goat anti-rabbit antibody conjugated to horseradish peroxidase (1:10,000 dilution; Invitrogen, Boston, MA) for 30 min. An anti-β-actin antibody conjugated to horseradish peroxidase (1:10,000 dilution; Sigma, St. Louis, MO) was used as the loading control. Membranes were developed with SuperSignal West Dura Extended Duration Substrate (Thermo Scientific, Waltham, MA) in accordance with the manufacturer's instructions and imaged on a myECL Imager (Thermo Scientific, Waltham, MA).

### Tissue histology and immunofluorescence microscopy.

Samples from the ileum and colon were fixed in 10% neutral formalin. Paraffin sections were stained with hematoxylin and eosin (H&E), periodic acid-Schiff, or immunofluorescence by standard protocols. The antibodies used were rabbit anti-muc2 and goat anti-lysozyme C (Santa Cruz Biotechnology, Dallas, TX). After antigen retrieval with citrate buffer, immunostaining for MUC2 and lysozyme was performed by overnight staining with a 1:100 dilution of the primary antibody at 4°C, followed by staining with a 1:200 dilution of the secondary antibody conjugated with Alexa Fluor 488 (Life Technologies, Grand Island, NY). The slides were mounted with SlowFade gold antifade reagent (Life Technologies, Grand Island, NY) after counterstaining with 4′,6-diamidino-2-phenylindole (DAPI).

### Induction of colitis and colitis assessment.

Acute colitis was induced in age-matched mice with 3% DSS (molecular mass of 36 to 50 kDa; MP Biomedicals, Solon, OH) in drinking water for 6 days. Body weight, stool consistency, and stool blood were recorded daily by a research technician (J.L.) blind to the study groups. Disease activity indices were calculated as described previously by averaging the scores for weight loss, stool consistency and bleeding, which were recorded on a scale of 0 to 4 (31). The histological assessment of colitis was performed by a trained pathologist (M.V.) blind to the treatment groups and scored by using the following criteria: architectural changes (0 = normal, 1 = mild focal abnormality, 2 = diffuse or multifocal mild to moderate abnormality, 3 = severe diffuse or multifocal abnormality); basal plasmacytosis and neutrophil/eosinophil infiltration (0 = none, 1 = mild, 2 = moderate, 3 = marked), crypt abscesses and crypt destruction (0 = absent, 1 = < 5% of crypts involved, 2 = 5 to 50% of crypts involved, 3 = >50% of crypts involved); and epithelial erosion or ulceration (0 = no erosion or ulceration, 1 = superficial erosion with epithelial repair, 2 = moderate ulceration with epithelial repair, 3 = ulcer with granulation tissue and no epithelium).

### Explant culture and IL-6 ELISA.

A small segment of distal colon was washed in PBS supplemented with 50 mg/ml gentamicin. The explant was cultured in 24-well plates containing RPMI 1640 medium supplemented with penicillin and streptomycin. After 24 h, the supernatant was collected, stored at −20°C, and later analyzed for interleukin-6 (IL-6) production with a Duoset mouse IL-6 enzyme-linked immunosorbent assay (ELISA) kit (R&D Systems, Minneapolis, MN). All other cytokines were measured by customized Luminex mouse magnetic bead assay according to the manufacturer's recommendations (R&D Systems, Minneapolis, MN).

### Luminal and mucosal microbial analysis.

Luminal samples comprised fecal pellets collected from the distal portion of the excised colon. Mucosal samples comprised the tissues from the same distal region and were collected after flushing the colon twice with PBS. Luminal fecal material and mucosal tissues from the distal portion of the ileum were collected in the same manner. Genomic DNA was extracted from the lumen and mucosa of the distal colon and ileum with a QIAamp stool and genomic DNA isolation kit (Qiagen, Valencia, CA). The 16S rRNA gene copy number as an estimate of the total bacterial number and individual bacterial groups in the lumen and mucosal samples was determined by qPCR with universal and group-specific primers as described above and previously (32). Analyses for microbial richness, composition, and diversity were conducted by Second Genome Inc. (San Francisco, CA) as described in the supplemental material.

### T-RF length polymorphism (T-RFLP).

Genomic DNA from luminal or stool samples was amplified with broad-range forward primer 8F (labeled with 6-carboxyfluorescein dye at the 5′ end) and reverse primer 1492R with GoTaq DNA polymerase (Promega, Madison, WI). The amplified PCR product was purified and digested by the MspI restriction enzyme (Promega, Madison, WI). The restriction digestion product was mixed with a GeneScan 1200 LIZ size standard (Life Technologies, Grand Island, NY) and deionized formamide. The length and intensity of terminal restriction fragments (T-RF) were determined on an ABI 3730 capillary sequencer (Life Technologies, Grand Island, NY) and analyzed with peak scanner software (Life Technologies, Grand Island, NY). The T-RF profiles were subjected to principal-component analysis (PCA) with the Excel add-in Multibase package (NumericalDynamics.Com, Tokyo, Japan).

### Statistical analyses.

Data were expressed as the mean ± the standard error of mean and analyzed in GraphPad Prism 6. Statistical significance was assessed with Student's *t* test, unless stated otherwise. For more than two groups, analysis of variance (ANOVA) with Tukey's *post hoc* test was used. Effects of time and genotype in DSS exposure experiments were determined by two-way ANOVA and Tukey's *post hoc* test. Statistical significance of microbiota sequence comparisons at various taxonomic levels was determined by Student's *t* test, followed by Benjamini-Hochberg adjustment for a false-discovery rate (FDR) of 10%. The Pearson correlation coefficient and significance were determined with IBM SPSS Statistics 22 software. Heat maps were constructed with CIMminer (Genomics and Bioinformatics Group, Laboratory of Molecular Pharmacology, Center for Cancer Research, National Cancer Institute). Differences were considered significant when the *P* value was <0.05.

### Nucleotide sequence accession number.

The sequences obtained in this study have been submitted to the European Bioinformatics Institute database under accession number PRJEB8110.

## RESULTS

### Villin-TLR4 mice display increased invasion by intestinal microbes.

**FIG 1:**
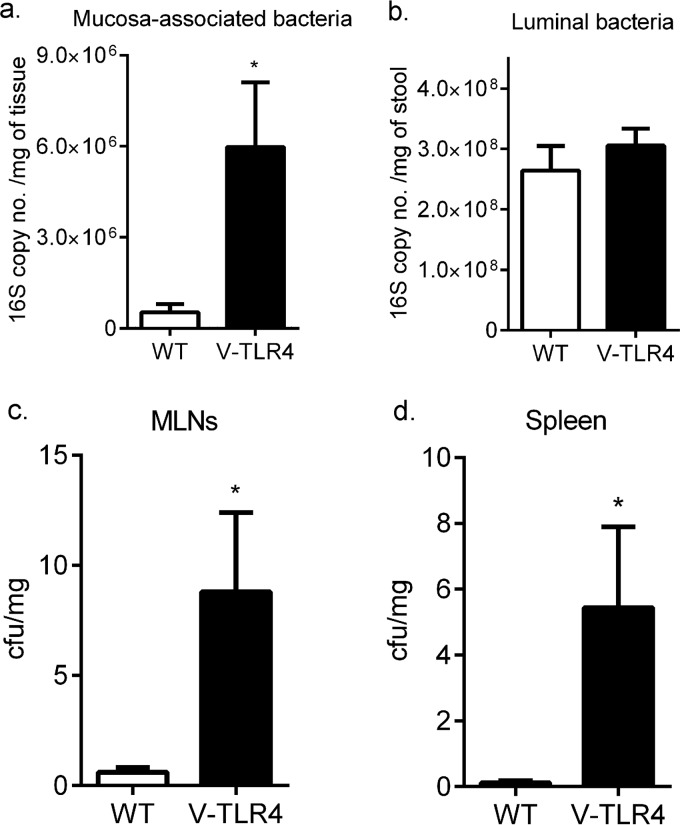
Increased mucosa-associated bacteria and bacterial invasion in villin-TLR4 mice. The 16S rRNA gene copy number of total bacteria per milligram of respective sample was determined for the mucosa (a) and lumen (b) of the distal colons of villin-TLR4 and WT littermates. Bacterial CFU per milligram of sample were counted by averaging the total number of colonies formed after aerobic and anaerobic culture of MLNs (c) and spleen tissue (d) from villin-TLR4 and WT littermate mice. Mean values ± the standard errors of the means of six to eight mice per group are shown. *, *P* < 0.05 by Student's *t* test.

In earlier work, we found that TLR4^−/−^ mice had increased bacterial translocation with DSS-mediated epithelial injury but not at the baseline (23). We have previously reported increased fecal IgA levels at the baseline in the context of increased epithelial TLR4 expression (26). Thus, we asked whether increased TLR4 signaling in the epithelium affected the total bacterial count in the distal colon and translocation in the absence of injury. We examined the quantity of the total bacteria in the intestinal mucosa and the lumen of villin-TLR4 and WT littermate mice by RT-qPCR of the 16S rRNA operon. We found approximately 10-fold greater mucosa-associated bacterial loads in villin-TLR4 mice than in WT littermates (Fig. 1a). However, in the colonic lumen, the counts of total bacteria and of representative bacterial groups (including Bacteroides, Lactobacilli, Ruminococcus, Clostridium leptum, Clostridium coccoides, and Prevotella) did not differ between villin-TLR4 and WT littermates (Fig. 1b; see Fig. S1 in the supplemental material). Given the greater bacterial density in the mucosa than in the lumen of villin-TLR4 mice, we asked whether there was an increase in bacterial translocation to the MLNs or spleen. Aerobic and anaerobic bacterial cultures of MLNs and spleen showed significantly more bacterial translocation in villin-TLR4 mice than in their WT littermates (Fig. 1c and d). Our data demonstrate that intestinal epithelium-specific TLR4 signaling promotes an increase in mucosa-associated bacteria and an associated increase in the translocation of intestinal bacteria systemically.

### Constitutive TLR4 signaling alters the luminal and mucosa-associated microbiota.

**FIG 2:**
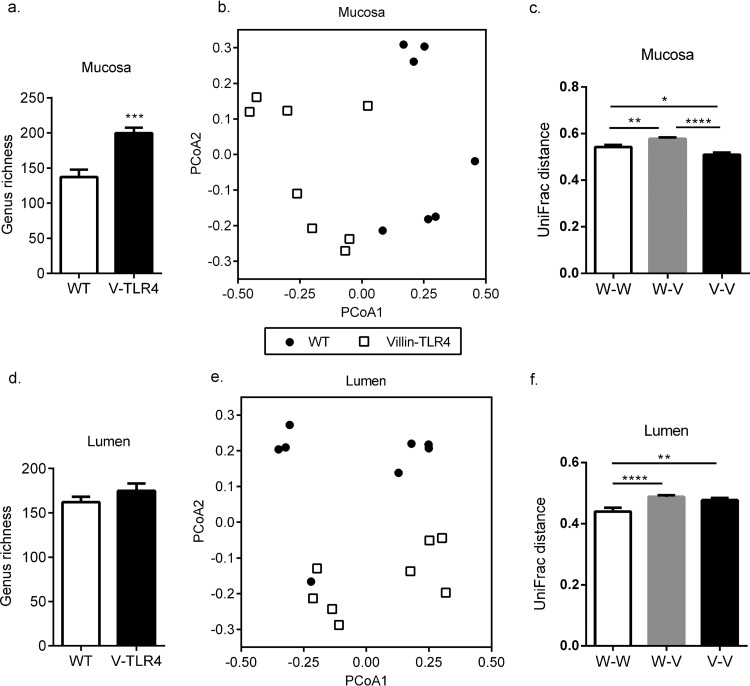
Epithelium-specific TLR4 expression changes the composition of the colon microbiota. To calculate bacterial richness, total numbers of OTUs at the genus level were determined by Illumina 16S rRNA gene sequencing of mucosa (a) and lumen (d) samples from the distal colons of villin-TLR4 and WT littermates (***, *P* < 0.001 by Student's *t* test). PCoA based on unweighted UniFrac distances between OTUs detected in the mucosa (b) and lumen (e) were used to generate ordination plots for viewing of the relative positioning of villin-TLR4 (open squares) and WT (filled circles) littermate mice in two dimensions. The values in parentheses indicate the percent variation explained by the axis. Mean UniFrac distances for each WT mouse versus every other WT mouse (W-W), each WT mouse versus every villin-TLR4 mouse (W-V), and each villin-TLR4 mouse versus every other villin-TLR4 mouse (V-V) are plotted for mucosal (c) and luminal (f) samples (*, *P* < 0.05; **, *P* < 0.01; ****, *P* < 0.0001 by one-way ANOVA and Tukey's *post hoc* test). Mean values ± the standard errors of the means of seven or eight mice per group are shown.

Given that villin-TLR4 mice had increased bacterial density in the colon mucosa, as well as increased bacterial translocation, we reasoned that villin-TLR4 mice might possess an altered microbiota in terms of the composition or relative abundance of various bacterial clades. To clearly define the effect of increased epithelial TLR4 signaling on the microbiota, we controlled for microbiota variations caused by factors such as maternal transmission, housing, and diet by using littermate mice. First, we analyzed the richness of the luminal and mucosal microbiota in the colons of villin-TLR4 and WT littermates. The overall richness of the mucosa-associated microbiota was significantly higher in the colons of villin-TLR4 mice than in those of their WT littermates (Fig. 2a). However, we did not observe any significant difference in the overall richness of the colonic luminal microbiota between the two groups (Fig. 2d). Next we wanted to identify the bacterial clades from the phylum to the genus level of taxonomy that are different either in their presence or absence or in their relative abundance in the setting of intestinal epithelium-specific constitutive TLR4 signaling. After adjustment for the FDR, several significant differences in bacterial presence and, to a lesser extent, abundance were observed between colonic luminal and mucosal samples of villin-TLR4 and WT littermates at all levels of bacterial taxonomy (Table 1). At the phylum level, significant changes in Fusobacteria, Firmicutes, and Proteobacteria were observed in the colonic mucosa of villin-TLR4 mice compared to that of their WT littermates. However, this change was not observed in luminal samples. The proportions of three bacterial families belonging to the phylum Firmicutes and one family belonging to Actinobacteria were significantly higher in the mucosa of villin-TLR4 mice. Three other families belonging to the phyla Fusobacteria, Firmicutes, and Gammaproteobacteria were present in lower proportions in the mucosa of villin-TLR4 mice. However, Lachnospiraceae was the only family whose increase in the mucosa-associated bacteria was also reflected in the lumen of villin-TLR4 compared with that of WT littermate mice. Of the 2,260 operational taxonomic units (OTUs) found in the mucosal samples, 92 and 177 differed significantly in their abundance and proportions, respectively, in villin-TLR4 and WT mice (see Tables S1 and S2 in the supplemental material). Similarly, the numbers of OTUs that differed between the luminal samples were 38 and 54, respectively (see Tables S3 and S4). However, very few of these OTUs passed the criterion of an FDR of <0.1 (see Tables S1 to S4).

**TABLE 1:** Bacterial differences observed between villin-TLR4 and WT littermates at various taxonomic levels^*a*^

Phylum	Class	Order	Family	Genus
**Firmicutes** (↑)	**Clostridia** (↑)	Lactobacillales (↓)		
		**Clostridiales** (↑)	**Lachnospiraceae** (↑+)	***94otu1346*** (↑)
				***94otu19924*** (↑+)
				**Blautia** (+)
			**Ruminococcaceae** (↑)	***94otu11945*** (↑)
				**Faecalibacterium** (↑)
			**Mogibacteriaceae** (↑)	
			***91otu5089*** (↓)	
			Veillonellaceae	**Dialister** (+)
				**Phascolarctobacterium** (↑)
Bacteroidetes	**Flavobacteria** (↓)	**Flavobacteriales** (↓)		
**Proteobacteria** (↓)	**Gammaproteobacteria** (↓)	**Enterobacteriales** (↓)	**Enterobacteriaceae** (↓)	
		**Pseudomonadales** (↓)		
	**Betaproteobacteria** (−)	**Burkholderiales** (−)		
	Alphaproteobacteria	**Rhodospirillales** (↓)		
Actinobacteria	**Coriobacteria** (↓+)	**Coriobacteriales** (↓+)	**Coriobacteriaceae** (↑)	
**Fusobacteria** (↓)	**Fusobacteria** (↓)	**Fusobacteriales** (↓)	**Fusobacteriaceae** (↓)	**Fusobacterium** (↓)

a The symbol ↑ or ↓ indicates an increase or decrease in villin-TLR4 mucosa relative to WT mucosa. The symbol + or − indicates an increase or decrease in villin-TLR4 lumen relative to WT lumen. Bold indicates a significant difference in presence or absence between the two groups (*P* < 0.05; FDR, <0.1). Underlining indicates a significant difference in relative abundance between the two groups (*P* < 0.05; FDR, <0.1).

Principal-coordinate analysis (PCoA) of the colonic mucosal microbiota using weighted UniFrac distances (based on microbial abundance) did not separate the samples on the basis of genotype (see Fig. S2a in the supplemental material). Similar results were obtained with luminal samples (see Fig. S2a in the supplemental material). However, when luminal and mucosal samples were analyzed by using the unweighted UniFrac metric (based on microbial presence or absence), we observed separate clustering of villin-TLR4 and WT mice (Fig. 2b and e). Furthermore, Adonis testing based on the randomized Monte Carlo permutation test was used to determine the statistical significance of microbiota differences between the two genotypes. The Adonis test performed on bacterial presence/absence validated that both the lumen (*P* = 0.011) and the mucosa (*P* = 0.001) of villin-TLR4 mice were associated with a significant difference between their microbiota and that of their WT littermates. A greater UniFrac distance between mice with different genotypes than between mice of the same genotype indicates that the microbiotas of the two genotypes are different (15, 33). Average UniFrac distances were significantly greater between WT and villin-TLR4 mice than the distances between WT and other WT mice and between villin-TLR4 and other villin-TLR4 mice (Fig. 2c and f). These results demonstrate that the microbiota of villin-TLR4 mice differs from that of their WT littermates in composition not only in the colonic mucosa but also in the lumen.

### TLR4 overexpression disrupts epithelial barrier function.

**FIG 3:**
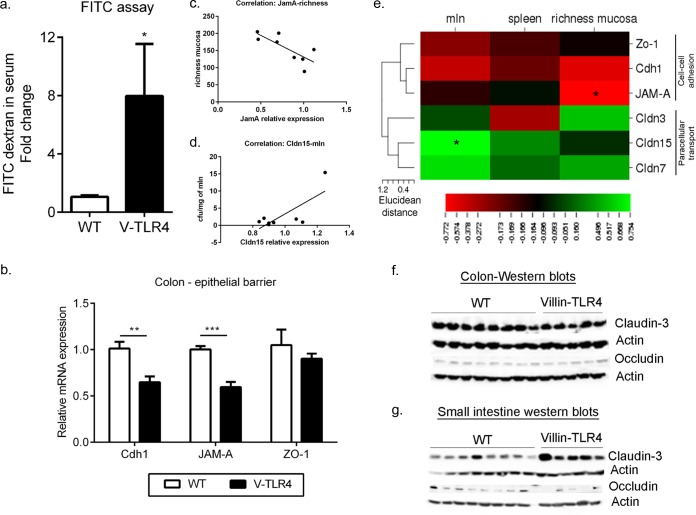
Compromised intestinal barrier and transport functions in villin-TLR4 mice promote bacterial dissemination. (a) Intestinal mucosal permeability was measured by 4-kDa FITC-dextran levels in the serum of villin-TLR4 and WT mice. Results represent the mean ± the standard error of the mean of fold changes in FITC-dextran levels relative to the WT (seven to nine mice per group). (b) Relative mRNA expression levels of epithelial barrier genes in the colonic ECs of villin-TLR4 and WT mice. Results represent the mean ± the standard error of the mean of relative mRNA expression normalized to GAPDH and β-actin (five mice per group). (c) Correlation between relative *JAM-A* mRNA expression levels and mucosal bacterial richness as determined by bacterial 16S rRNA gene sequencing (*r* = −0.77; *P* < 0.05). (d) Correlation between relative Cldn15 mRNA expression levels and bacterial invasion as determined by CFU counts in MLN cultures of villin-TLR4 and WT littermates (*r* = −0.75; *P* < 0.05). (e) Pearson correlation between epithelial barrier genes and markers of bacterial translocation (bacterial richness in mucosa and bacterial CFU counts in MLN and spleen tissues, respectively) showed a mostly negative correlation with cell-cell adhesion genes and a positive correlation with junctional paracellular transporters. The Pearson correlation coefficients (*r* values) of these variables were mapped by a one-matrix heat map with CIMminer. The scale bar, from left to right, represents negative correlations in red to positive correlations in green. Epithelial barrier genes were clustered by Euclidean distance. *, *P* < 0.05; **, *P* < 0.01; ***, *P* < 0.001 by two-tailed Student *t* test. (f, g) Western blot analysis of claudin-3 and occludin protein levels in colonic and small IECs of WT and villin-TLR4 littermate mice. β-Actin was used as a protein loading control for each Western blot gel.

We wanted next to understand the mechanism by which epithelial TLR4 altered the microbiota composition and invasion. We hypothesized that compromised intestinal permeability in villin-TLR4 mice may play a role in increased bacterial dissemination. We investigated the epithelial permeability of villin-TLR4 and WT littermates by oral administration of 4-kDa FITC dextran and measured the levels of FITC-dextran in their serum after 4 h. Significantly higher levels of FITC-dextran were observed in villin-TLR4 mice than in their WT littermates (Fig. 3a). These data suggest that epithelial function is altered by increased epithelial expression of TLR4.

In order to examine the mechanism by which TLR4 might be altering epithelial permeability, we asked whether villin-TLR4 mice had altered expression of genes involved in intestinal epithelial barrier and transport functions. To address this, we isolated IECs from villin-TLR4 and WT littermate mice and used real-time RT-qPCR to examine genes involved in junctional epithelial cell-cell adhesion, junctional paracellular transport, and transcellular transport (34). Of these, the expression of epithelial cell-cell adhesion genes (35, 36), including those for junctional adhesion molecule A (*JAM-A*) and cadherin-1 (*Cdh1*), was significantly lower in colonic ECs of villin-TLR4 mice than in those of WT mice (*P* < 0.05) (Fig. 3b). Additionally, the expression of the gene for zonula occludens 1 (*ZO-1*) (37) was lower in villin-TLR4 mice, although not significantly so (Fig. 3b). However, no significant changes were observed in the genes for claudins (*Cldn3*, *Cldn7* and *Cldn15*), which have been shown to directly regulate epithelial permeability by forming paracellular channels (38, 39) (see Fig. S3a in the supplemental material). To confirm our observations from gene expression studies, we performed Western blot analysis of EC lysates with antibodies specific for Cldn3 and occludin. Consistent with our findings from gene expression studies, we did not observe any differences in Cldn3 expression between colonic ECs of the villin-TLR4 and WT littermates (Fig. 3f). Similarly, the expression of the gene for occludin was not different in the colonic ECs of the two genotypes. No significant differences were observed between villin-TLR4 and WT littermate mice in the expression of the genes for any of the junctional proteins in the small intestinal ECs (see Fig. S3c in the supplemental material). However, Western blot analysis of small intestinal EC lysate indicated that Cldn3 gene expression was greater in villin-TLR4 mice than in WT mice, whereas occludin gene expression was reduced in the small intestine ECs of villin–TLR4 mice (Fig. 3g). For genes encoding transcellular transporters, we found a very significant increase in the expression levels of ion transport gene *Clca4* (chloride channel calcium activated 4), a biomarker of inflammatory bowel disease (40), in the colonic ECs of villin-TLR4 mice (see Fig. S3d in the supplemental material). We also observed a decrease in the gene for carbonic anhydrase 4 (*Car4*), which is important for chloride ion homeostasis (41), in villin-TLR4 mice relative to WT littermates. Similarly, we observed a significant decrease in *car4* expression and an upregulation of *Clca4* expression in the small intestinal ECs of villin-TLR4 mice relative to their WT littermates (see Fig. S3e in the supplemental material). Expression of aquaporin 4 (*Aqp4*) did not differ between villin-TLR4 and WT littermates in either region of the intestine (see Fig. S3b in the supplemental material). Taken together, the above findings suggest that intestinal epithelium-specific TLR4 signaling regulates specific barrier and transport functions of the host.

To identify whether increased bacterial invasion in the colonic mucosa of villin-TLR4 is associated with decreased epithelial barrier formation, Pearson's correlation analysis was performed between variables representing markers for bacterial invasion and the expression of epithelial barrier function genes. Indeed, a negative correlation was found between the bacterial CFU counts in the MLNs and spleen, mucosal richness, and relative expression levels of the genes for colonic junctional cell-cell adhesion proteins (*ZO-1*, *JAM-A*, and *Cdh1*, respectively) (Fig. 3e). However, the correlation coefficient was significant only for mucosal richness and *JAM-A* (Fig. 3c). Interestingly, the correlation between variables for bacterial invasion and claudins, the junctional paracellular transporters, was positive, with a significant coefficient of correlation between *Cldn15* and bacterial CFU counts in MLNs (Fig. 3d and e). Although *Cldn15* was not significantly different between the two genotypes, a higher correlation was observed because villin-TLR4 mice that had less bacterial invasion also had lower expression of *Cldn15*. These results suggest that increased epithelial permeability is positively associated with increased systemic bacterial dissemination.

### Constitutive TLR4 signaling alters expression of AMP in IECs.

**FIG 4:**
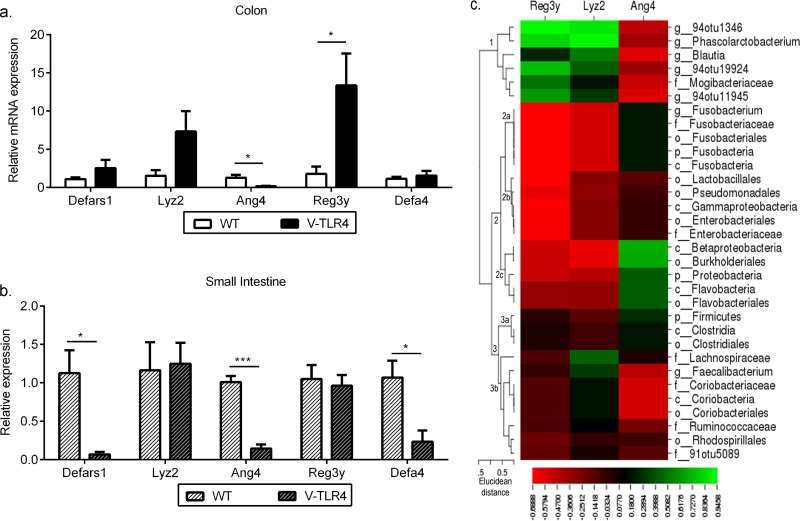
Expression of AMP genes in the colon and small intestine is distinctly regulated by epithelial TLR4. Relative mRNA expression levels of AMP genes in ECs isolated from the colons (a) and small intestines (b) of villin-TLR4 and WT mice are shown. Results represent the mean ± the standard error of the mean of relative mRNA expression normalized to GAPDH and β-actin. *, *P* < 0.05; ***, *P* < 0.001 by two-tailed Student's *t* test (four or five mice per group). (c) Pearson coefficients of correlation between selected AMP genes and bacterial OTUs were both negative and positive. The Pearson correlation coefficients (*r* values) obtained for each AMP gene and selected bacterial OTUs were mapped by a one-matrix heat map with CIMminer. The scale bar, from left to right, represents negative correlations in red to positive correlations in green. Bacterial OTUs were clustered by Euclidean distance. Clusters and subclusters are labeled at their nodes.

Expression of antimicrobial peptides (AMP) is triggered by bacterial colonization, infection, and inflammation (42–44). On the basis of our findings of increased mucosa-adherent bacteria in villin-TLR4 mice, we hypothesized that these mice will display altered expression of AMP genes in the intestinal epithelium. We measured the relative expression levels of AMP genes in the small intestines and colonic ECs of villin-TLR4 and WT littermates. We observed a significant increase in the levels of *Reg3g* expression in colonic ECs of villin-TLR4 mice, and a similar trend was observed in the gene for lysozyme 2 (*Lyz2*). However, we found an opposite trend in the gene for angiogenin 4 (*Ang4*) in the colonic epithelium of villin-TLR4 mice (Fig. 4a). Our observation of increased *Reg3g* expression in villin-TLR4 mice is consistent with previously published studies wherein increased *Reg3g* expression occurred in response to LPS, the ligand for TLR4, in germfree and antibiotic-treated mice, suggesting that TLR4 regulates the expression of *Reg3g* (45, 46). Another possibility is that *Reg3g* expression increased in response to an increased bacterial load in the colonic mucosa of villin-TLR4 mice.

To determine whether the changes observed in the mucosal microbiota are associated with antimicrobial gene expression in the ECs of the colon, Pearson's correlation analysis of these parameters was performed (for R values, see Table S5 in the supplemental material). Only bacterial clades that differed between villin-TLR4 and WT mice from Table 1 were considered for this analysis. On the basis of the Pearson correlation coefficient values obtained for each bacterial clade and antimicrobial gene, the bacterial clades separated into three clusters by Euclidean distance (Fig. 4c). Cluster 1, which consisted of bacterial OTUs that increased in villin-TLR4 mice, correlated positively with *Reg3g* and *Lyz2* and negatively with *Ang4*. Within cluster 2, which consisted of bacterial OTUs that decreased in villin-TLR4 mice, a correlation trend that was the opposite of that of cluster 1 was observed between OTUs and AMP genes for subclusters 2a and 2c. Subcluster 2b correlated negatively with *Reg3g* and *Lyz2* and was unaffected by *Ang4*. Cluster 3 consisted of bacteria that increased or decreased in villin-TLR4 mice. No specific trend was observed in this cluster, with subcluster 3a correlating positively with *Ang4* and remaining unaffected by the other two AMP genes. Subcluster 3b mostly showed an association that was the opposite of that of subcluster 3a, with five of the eight bacterial OTUs correlating negatively with *Ang4*. These results reinforce the notion obtained from previous studies (47) that different AMP genes may act synergistically or antagonistically to regulate the microbiota composition.

Contrary to the observations in the colonic epithelium, we found a general trend of decreased expression of AMP genes in the small intestines of villin-TLR4 mice. Primarily, the genes coding for α-defensins (*defars1*, *Defa4*) and *Ang4* were significantly lower in the small intestinal ECs of villin-TLR4 mice (Fig. 4b). To determine if decreased expression of AMP genes in the small intestine has any effect on the ileum microbiota, we examined the total quantities of bacteria in the mucosa and luminal regions of the ilea of villin-TLR4 and WT littermate mice by RT-qPCR of the 16S rRNA operon. Interestingly, we did not observe any significant differences between the total bacterial loads in the lumen and mucosa of the villin-TLR4 mouse ileum and those of the WT ileum (see Fig. S4 in the supplemental material). A PCA plot generated by accounting for the relative abundance of bacterial taxonomic groups obtained by T-RFLP did not showed any separation of cohoused villin-TLR4 and WT littermates (see Fig. S4 in the supplemental material).

Our results suggest that epithelial TLR4 regulates AMP expression diversely in the small intestine and colon. While AMP expression can influence the microbiota composition and abundance or vice versa in the colon, similar regulation does not appear to occur in the small intestine.

### Epithelial TLR4 signaling regulates cell lineage commitment in the small intestine.

**FIG 5:**
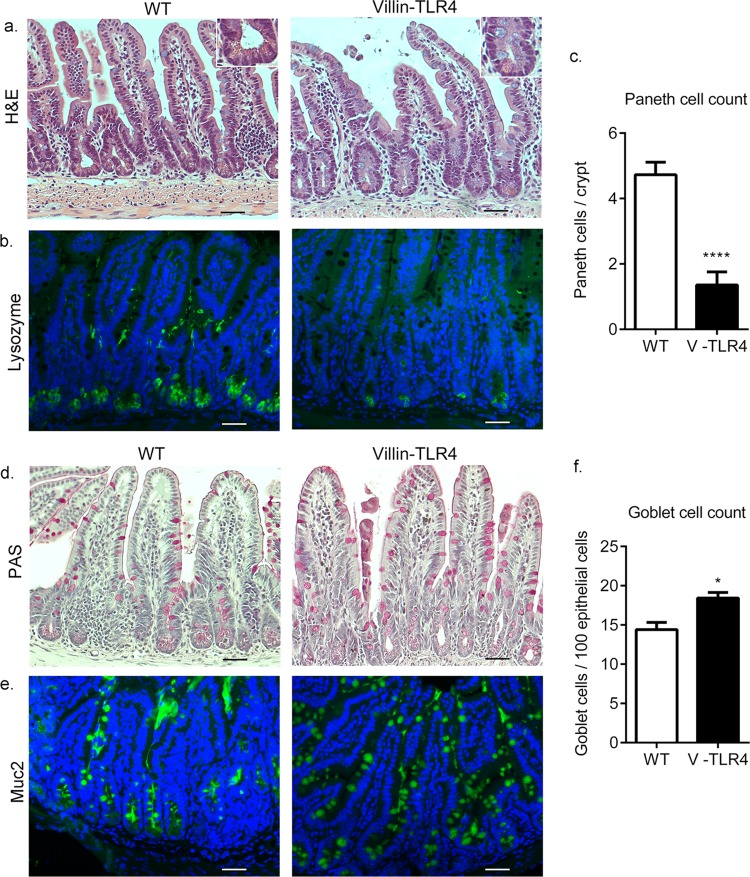
TLR4 regulates EC differentiation in the small intestine. (a) Histological images of ileum tissues from WT and villin-TLR4 littermate mice at 8 weeks of age. The inset shows a lower number of Paneth cells per crypt in villin-TLR4 mice than in WT mice. (b) Reduced lysozyme (green) staining in the ileum tissue of villin-TLR4 mice. (c) Graph of the number of Paneth cells (mean ± the standard error of the mean) in ileal sections of small intestine tissue as determined by H&E staining. Higher numbers of goblet cells in the ileum tissue of villin-TLR4 mice than in that of WT mice are indicated by periodic acid-Schiff (PAS) (d) and Muc2 (green) immunofluorescence (e) staining. (f) Graph of goblet cell numbers (mean ± the standard error of the mean) determined in PAS-positive cells in ileum tissue sections from WT and villin-TLR4 mice. For immunofluorescent images (b, e), nuclei were stained with DAPI (blue). Images are representative of four mice per group. Scale bars, 200 μm. Magnification, ×20. *, *P* < 0.05; ****, *P* < 0.0001 by Student's t test.

TLR4 regulates the development of goblet cells in the small intestine (48). We have previously shown that constitutive epithelial TLR4 signaling affects the crypt-villus structure of the intestine (49). Given that Paneth and goblet cells are primary sources of AMP in the intestine, we asked whether constitutive expression of TLR4 affected the differentiation of these cell types in the small intestine and colon. Consistent with the decrease in α-defensin expression, we observed a significantly fewer Paneth cells (Fig. 5c) in the ilea of villin-TLR4 mice than in those of their WT littermates, as determined by H&E staining and lysozyme immunofluorescent staining (Fig. 5a and b). To examine goblet cell quantity, we stained for goblet cells with periodic acid-Schiff and immunofluorescent staining for MUC2 (Fig. 5d and e; see Fig. S5a and b in the supplemental material). We observed a small but statistically significant increase in the goblet cell count in the ilea (Fig. 5f) but not the colons of villin-TLR4 mice (see Fig. S5c in the supplemental material). Our results indicate that TLR4 signaling affects EC differentiation in the small intestine but not in the colon. These results further suggest that the increased mucosa-adherent bacteria and altered AMP expression in the colons of villin-TLR4 mice were not primarily a result of altered cell differentiation. On the other hand, altered AMP expression in the small intestine may be a result of TLR4-induced effects on Paneth and goblet cell differentiation. Our observations in this study are consistent with previously published studies wherein, by using transgenic mice ablated for Paneth cells, it was shown that although Paneth cells are required to limit bacterial translocation to MLNs and the spleen, no difference in the luminal bacterial load or distribution of bacteria along the crypt-villus axis occurs in these transgenic mice (44, 50). On the basis of these findings, we propose that the decreased AMP production in the small intestines of villin-TLR4 mice occurs primarily because of a reduced number of Paneth cells. This reduced AMP expression does not, however, appear to affect bacterial abundance or distribution in the small intestine.

### Cohousing with villin-TLR4 mice increases the sensitivity of WT mice to DSS-induced colitis.

**FIG 6:**
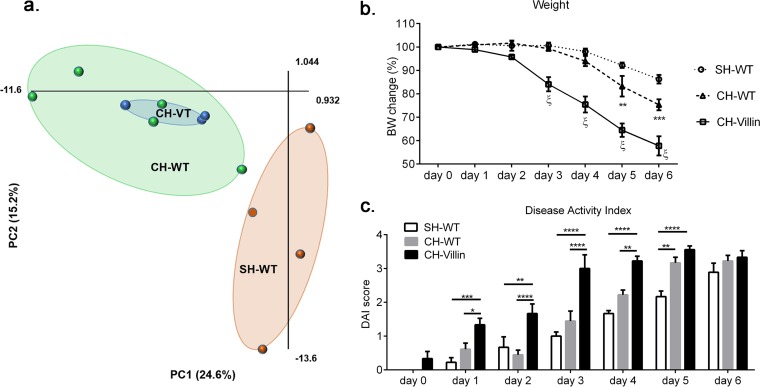
Increased severity of DSS-induced colitis in villin-TLR4 (CH-villin) mice is transmissible to CH-WT mice. (a) PCA plot showing the relative positioning of SH-WT (orange), CH-WT (green), and CH-villin (blue) mice based on the relative abundance matrix of T-RF obtained by T-RFLP analysis of the 16S rRNA gene. The two principal components (PC1 and PC2) explain 39.8% of the variance. (b) Percent body weight (BW) changes in CH-villin, CH-WT, and SH-WT mice that were given 3% DSS in drinking water for 6 days (ξ indicates that CH-villin mice were significantly different from both SH-WT and CH-WT mice [*P* < 0.0001], and asterisks indicate that CH-WT mice were significantly different from SH-WT [**, *P* < 0.01; ***, *P* < 0.001] by two-way ANOVA and Tukey's *post hoc* test). (c) DAI scores based on stool blood, weight loss, and stool consistency from day 0 to day 6 of DSS exposure (*, *P* < 0.05; **, *P* < 0.01; ***, *P* < 0.001; ****, *P* < 0.0001 by two-way ANOVA and Tukey's *post hoc* test).

We have previously shown that villin-TLR4 mice are more prone to DSS-induced colitis than their WT littermates (25). Thus, we questioned whether the altered microbiota of villin-TLR4 mice might play a role in the increased susceptibility of villin-TLR4 mice to colitis. To test this, we cohoused C57BL/6 WT mice with villin-TLR4 mice for 4 weeks and also housed C57BL/6 WT mice by themselves in a separate cage. Previously published studies have shown that 4 weeks of cohousing is sufficient for the transmission of microbiota between mice of different genotypes (29). Indeed, after cohousing, the stool microbiota of cohoused WT (CH-WT) mice was more similar to that of villin-TLR4 (CH-VT) than to that of separately housed WT (SH-WT) mice (Fig. 6a). At the end of 4 weeks, acute colitis was induced in SH-WT, CH-WT, and villin-TLR4 (CH-VT) mice by the addition of 3% DSS to the drinking water for 6 days. Similar to our previous observations, we observed greater weight loss in villin-TLR4 mice than in WT mice. Interestingly, at day 3, CH-WT mice started losing more weight than SH-WT mice. This weight loss effect was more pronounced at days 5 and 6 of DSS exposure, where CH-WT mice lost significantly more weight than SH-WT mice did (Fig. 6b). A similar trend was observed in the disease activity index (DAI), wherein villin-TLR4 mice had the highest DAI score, followed by CH-WT mice, and the lowest DAI score was that of SH-WT mice, especially early after DSS introduction (Fig. 6c). We also observed that almost all of the villin-TLR4 mice (five out of six) but none of the cohoused or singly housed WT mice died at day 6 from DSS exposure. Considering our earlier observation that the luminal and mucosa-associated microbiotas of villin-TLR4 and WT littermates are different, the findings from cohousing experiments suggest that both the microbiota and host TLR4 expression contribute to the induction of severe colitis in villin-TLR4 mice after DSS exposure.

**FIG 7:**
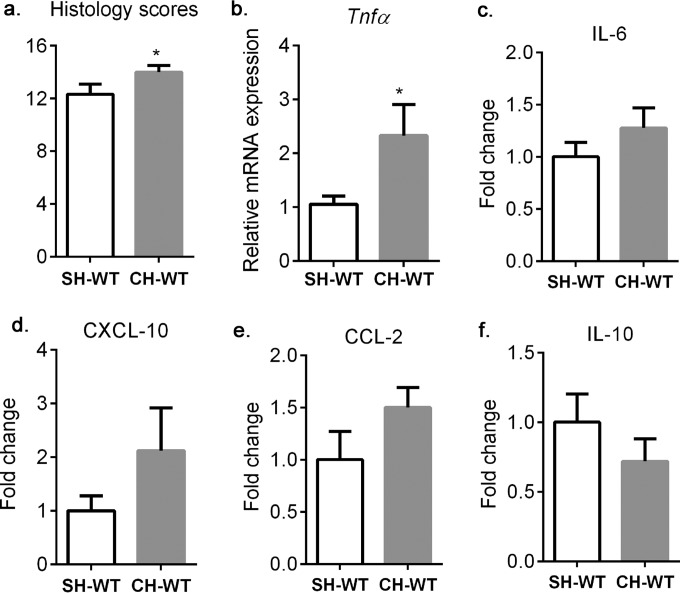
Cohousing with villin-TLR4 mice increases colonic inflammation in WT mice after DSS exposure. (a) Histological inflammatory scores of disease severity of SH-WT and CH-WT mice on day 6 of DSS treatment. (b) Relative levels of TNF-α mRNA expression in the proximal colons of SH-WT and CH-WT mice. (c to f) Fold changes in distal colon explant IL-6, CXCL-10, CCL2, and IL-10 protein secretion levels in CH-WT versus SH-WT mice. *, *P* < 0.05 by one-tailed Student *t* test. Mean values ± the standard errors of the means of five or six mice per group are shown.

On the basis of the above findings, we hypothesized that CH-WT mice will exhibit more colonic inflammation than SH-WT mice. Histologic observations based on architectural changes, basal plasmacytosis, neutrophil/eosinophil recruitment, acute cryptitis/crypt abscesses, crypt destruction, and epithelial erosion or ulceration showed signs of severe colonic inflammation in all of the groups. However, the colons of CH-WT mice were significantly more inflamed than those of SH-WT mice (Fig. 7a). Moreover, the expression of the gene for the inflammatory cytokine tumor necrosis factor alpha (TNF-α) was significantly greater in CH-WT mice than in SH-WT mice (Fig. 7b). Measurement of protein expression secreted by overnight culture of distal colon explants showed a notable trend toward greater IL-6, CXCL-10, and CCL2 secretion and lower IL-10 secretion in CH-WT mice than in SH-WT mice (Fig. 7c). The levels of gamma interferon, IL-12, IL-1β, and TNF-α were below the detection limit of the assay in most of the samples. To elucidate the mechanism behind the observed increased susceptibility to DSS in CH-WT mice, we assessed whether the barrier defects observed in villin-TLR4 mice are transmissible to CH-WT mice before DSS exposure. We did not observe any difference in bacterial translocation or FITC-dextran levels in serum between SH-WT and CH-WT mice (see Fig. S6 in the supplemental material). The data from these experiments demonstrated that although the microbiota of villin-TLR4 mice is transmissible to CH-WT mice, the barrier defects were not transmissible.

## DISCUSSION

Intestinal homeostasis depends on balanced innate immune responses and a healthy microbiota. We still do not know what constitutes a “healthy” microbiota, nor do we understand the interdependence of host signaling and cognate flora. If innate immune signaling is too weak, the host may be exposed to potential pathogens. If it is too vigorous, innate immune signaling may cause detrimental inflammation in the face of harmless commensals. In IBD, as either a cause or an effect of the disease process, epithelial expression of TLR4 is increased (19–21). In addition to IBD, TLR4 expression is increased in colitis-associated cancer and in some sporadic colon cancers (25, 49). We therefore wished to understand the impact of dysregulated epithelial TLR4 expression on the microbiota and on epithelial function.

Although *in vivo* diseases demonstrate TLR overexpression, most studies have used global knockouts or epithelial knockouts of innate immune receptors (or adapters) to address the role of innate immune signaling in the epithelium. In our model, we have turned up the volume of TLR signaling in the epithelium by expressing a constitutively active TLR4 gene in the epithelium. Here, by meticulously regulating confounding factors such as housing and maternal transmission commonly observed in microbiota studies, we demonstrated that the microbiota is altered in terms of quantity, composition, and richness in response to increased TLR4 signaling. The microbiota differences between villin-TLR4 and WT littermate mice were apparent in the mucosal lining and in the lumen and were transmissible. We also show that epithelial TLR4 signaling has differential, site-specific effects on EC differentiation, barrier formation, and AMP gene expression.

We found that numerous mechanisms involved in the regulation of the microbiota were impacted by TLR4 signaling. First, we found that epithelial barrier function was reduced in villin-TLR4 mice. We also found a positive correlation between lost barrier function and increased bacterial translocation. Second, we observed that Paneth cell numbers and alpha-defensin expression were decreased in the small intestines of villin-TLR4 mice. Paneth cells play a role in limiting the penetration of the mucosa by commensal and pathogenic bacteria (44). Thus, decreased Paneth cell number and function are likely to have a profound effect on bacterial invasion. Third, here we have reported that *Reg3g* and *Lyz2* expression is increased in the colons of villin-TLR4 mice, providing evidence that epithelial TLR4 signaling may differentially regulate AMP expression in the small intestine and colon. As indicated by the altered microbiota and increased number of mucosa-associated bacteria in *Reg3g*-deficient mice, *Reg3g* can modulate the composition of the mucosal microbiota and provide a first line of defense against mucosal association by bacteria (45). Correlation studies performed herein demonstrated a relationship between bacterial translocation and targeted epithelial barrier gene expression and also between specific alterations in the microbiota and antimicrobial gene expression in the colon. Although these studies do not prove causality, they permit a new view of host-microbial interdependence.

Our findings revealed higher levels of Fusobacteria and Proteobacteria and lower levels of Firmicutes in the colonic mucosa of villin-TLR4 mice than in that of WT littermate mice. Although villin-TLR4 mice are more susceptible to colitis, the microbial changes observed in these mice are the direct opposite of what is normally observed in IBD patients (51, 52). Here, it should be noted that although numerous gut microbiota studies have used murine models to gain an insight into pathological mechanisms of various diseases, direct comparison of mouse and human microbiotas is impractical because of numerous differences that exist between the human and murine gut microbiotas (53, 54). Despite this, we observed some interesting microbiota similarities between villin-TLR4 mice and IBD patients (55–57). As in IBD patients, we observed more mucosa-associated bacteria and Gram-positive Coriobacteriia and increased intestinal permeability in villin-TLR4 mice. Another potential reason for the observed reduction in potential pathogens in villin-TLR4 mice could be that upregulated expression of AMP genes in response to bacterial invasion and increased abundance of Coriobacteriia in the colons of villin-TLR4 lead to decreased numbers of other potential pathogens, including Fusobacteria and Proteobacteria, in the colon mucosa but an overgrowth of other species in the phylum Firmicutes.

Cohousing promotes the transmission of colonic microbiota between mice because of coprophagia. By T-RFLP analysis, we showed that the microbiota of CH-WT mice was more similar to that villin-TLR4 mice than to that of SH-WT mice. We also showed that C57BL/6 mice cohoused with villin-TLR4 mice develop more severe DSS colitis than singly housed C57BL/6 mice do. Recently Couturier-Maillard et al. reported that the microbiota associated with Nod2 deficiency gives rise to communicable yet reversible colitis (28). Similarly, it has been reported that the colitogenic properties of the microbiota from inflammasome-deficient mice are transferrable to CH-WT mice (29). On the basis of these findings, we believe that epithelial TLR4 signaling-induced changes in the microbiota are transmissible to WT mice and are responsible for increased susceptibility to DSS in CH-WT mice. We did not observe any signs of leaky gut in CH-WT mice. However, we observed an apparent increase in the proinflammatory chemokines CXCL-10 and CCL-2 and a decrease in the anti-inflammatory cytokine IL-10 in distal colon explants from cohoused mice. Although these differences between cohoused and SH-WT mice were not significant, we speculate that the differences in the microbiotas of these two groups lead to differential immune cell activation and recruitment, resulting in the observed differences in DSS susceptibility and the release of cytokines and chemokines.

Our studies cannot clearly prove cause and effect—does the microbiota change epithelial function, or is altered epithelial function responsible for a change in the microbiota? The experimental manipulation in this case is epithelial TLR4 expression. We observed location-specific effects of epithelial TLR4 expression on the microbiota, EC differentiation, barrier formation, and AMP gene expression. While the effect of TLR4 signaling on barrier and transport functions was observed in both the colon and small intestine, microbial dysbiosis was observed only in the colon and altered EC differentiation occurred only in the small intestine. The effect of increased TLR4 signaling on AMP gene expression was reversed in the small intestine and colon: in the colon, the AMP genes were generally upregulated, whereas in the small intestine, the AMP genes were downregulated. Thus, it seems likely that TLR4-induced alterations in the epithelium drive changes in the colonic microbiota. Once these changes are in place, the microbiota contributes to disease pathogenesis, as witnessed by our acute colitis cohousing experiment. We have previously shown that TLR4 signaling does regulate the expression of defensins both *in vitro* and *in vivo* (58). The results of this study imply that TLR4 expression regulates defensin expression by affecting Paneth cell differentiation in the small bowel. Paneth cells play a role in the limitation of bacterial translocation. Thus, it appears that reduced Paneth cell population size, coupled with a defective epithelial barrier, played a role in systemic bacterial translocation in villin-TLR4 mice.

On the basis of our findings, we can conclude that increased epithelial TLR4 signaling has clear effects on epithelial function and the microbiota. In states like IBD, wherein TLR4 expression does not shut off, as would be the case in a self-limited colitis, changes in epithelial function and the microbiota may contribute to the perpetuation of inflammation. As we learn more about microbiota composition, we will identify patterns of pathogenic, proinflammatory flora in the hope that this can be returned to normalcy. Our data also suggest that targeting of TLR4 may have a beneficial effect by restoring epithelial function and changing the microbiota.

## Supplementary Material

Supplemental material
