# Supplementary material for: Intestinal Epithelial Toll-Like Receptor 4 Signaling Affects Epithelial Function and Colonic Microbiota and Promotes a Risk for Transmissible Colitis
Source: Infect Immun. 2016 Feb 24;84(3):798–810. doi: 10.1128/IAI.01374-15 (PMC4771346; doi:10.1128/IAI.01374-15)
Supplement: Supplemental material [file supp_84_3_798__index.html]

Intestinal Epithelial Toll-Like Receptor 4 Signaling Affects Epithelial Function and Colonic Microbiota and Promotes a Risk for Transmissible Colitis — Supplemental material 

# Intestinal Epithelial Toll-Like Receptor 4 Signaling Affects Epithelial Function and Colonic Microbiota and Promotes a Risk for Transmissible Colitis

## Supplemental material

- Supplemental file 1 -

  Supplemental methods. Fig. S1. Quantification of bacterial groups in the distal lumen of villin-TLR4 and WT littermates. Fig. S2. Relative positioning of villin-TLR4 and WT littermate mice in two dimensions. Fig. S3. Epithelial gene expression in villin-TLR4 and WT littermate mice. Fig. S4. Bacterial composition in the ileum region of villin-TLR4 and WT littermate mice. Fig. S5. Goblet cell differentiation in villin-TLR4 and WT littermates. Fig. S6. Bacterial translocation and intestinal permeability in cohoused wild-type (CH-WT) and separately housed wild-type (SH-WT) mice.

  PDF, 382K
- Supplemental file 2 -

  Table S1. Mucosa-associated bacterial OTUs that differed between villin-TLR4 and WT littermates in relative abundance.

  XLSX, 18K
- Supplemental file 3 -

  Table S2. Mucosa-associated bacterial OTUs that differed between villin-TLR4 and WT littermates in their presence or absence.

  XLSX, 21K
- Supplemental file 4 -

  Table S3. Luminal bacterial OTUs that differed between villin-TLR4 and WT littermates in relative abundance.

  XLSX, 14K
- Supplemental file 5 -

  Table S4. Luminal bacterial OTUs that differed between villin-TLR4 and WT littermates in their presence or absence.

  XLSX, 14K
- Supplemental file 6 -

  Table S5. R values for Pearson's correlation analysis between mucosal microbiota and antimicrobial gene expression.

  XLSX, 15K
