# Supplementary material for: Intestinal Epithelial Toll-Like Receptor 4 Signaling Affects Epithelial Function and Colonic Microbiota and Promotes a Risk for Transmissible Colitis
Source: Infect Immun. 2016 Feb 24;84(3):798–810. doi: 10.1128/IAI.01374-15 (PMC4771346; doi:10.1128/IAI.01374-15)
Supplement: Supplemental material [file IAI.01374-15_zii999091620so1.pdf]

Supplementary material for **Dheer, R et al**; "Intestinal epithelial TLR4 signaling affects epithelial function, colonic microbiota and promotes risk for transmissible colitis"

Primers used in the study:

Bacteria/Gene	Forward primer 5′-3′end	Reverse primer 5′-3′end	Reference
Total Bacteria	actcctacgggaggcagcagt	attaccgcggctgctggc	1
Lactobacillus	agcagtagggaatcttcca	caccgctacacatggag	1
C.coccoides	actcctacgggaggcagc	gcttcttagtcaggtaccgtcat	1
Bacteroides	ggttctgagaggaggtccc	gctgcctcccgtaggagt	1
C.leptum	gttgacaaaacggaggaagg	gacgggcggtgtgtacaa	1
Bifidobacterium	ctcctggaaacgggtggt	gctgcctcccgtaggagt	1
Ruminococcus	cagcagccgcggtaata	cccacacctagtaatcatcgtt	1
Prevotella	cagcagccgcggtaata	ggcatccatcgtttaccgt	1
Cldn3	aagccgaatggacaaagaa	ctggcaagtagctgcagtg	2
Cldn15	cagcttcggtaaatatgcca	cagtgggacaagaaatggtg	2
Cldn7	agggtctgctctggtcctt	gtacgcagctttgctttca	2
ZO-1	aggacaccaaagcatgtgag	ggcattcctgctggttaca	2
Cdh1	tccttgttcggctatgtgtc	ggcatgcacctaagaatcag	2
JAM-A	accctccctcctttccttac	ctaggactcttgcccaatcc	2
Car4	ctccttcttgctctgctg	gactgctgattctcctta	3
Clca4	acatggaccggcctttctac	cgacatctcctcgacacaca	4
Aqp4	ttggacccgcagttatcatg	gcgacgtttgagctccacat	5
Defa4	ccaggggaagatgaccaggctg	tgcagcgacgatttctacaaaggc	6
Defa-rs1	caccacccaagctccaaatacacag	atcgtgaggaccaaaagcaaatgg	6
Lyz2	ggctggctactatggagtcagcctg	gcattcacagctcttggggttttg	6
Ang4	gctgggtctggttgtgattcc	aggcgaggttagctttctttcc	6
Reg3g	ttcctgtcctccatgatcaaa	catccacctctgttgggttc	7
Tff3	ttgctgggtcctctgggata	gccggcaccatacattgg	8
Muc2	aacgatgcctacaccaaggtc	actgaactgtatgccttcctca	4
Tnf-a	cgtggaactggcagaagagg	ggaatgagaagaggctgagacat	9
b-Actin	ggctgtattcccctccatcg	ccagttggtaacaatgccatgt	6
Gapdh	ccatcaccatcttccaggag	gtggttcacacccatcacaa	7

Supplementary methods

Analysis of mucosal and luminal bacteria:

Variable region 4 of bacterial 16S rRNA operon was amplified from the luminal and mucosal genomic DNA of villin-TLR4 and WT littermates using fusion primers incorporating sequences for Illumina flow cell adapters (San Diego, CA) and indexing barcodes. All samples except one mucosal sample from WT group met the post-PCR quantification minimum set by the second

genome Inc. and were advanced for purification and 250 cycles of sequencing on the MiSeq instrument. The sequences were quality filtered and demultiplexed using QIIME[10] and custom scripts. The sequences were then clustered at 97% similarity by uclust (closed reference OTU picking) and representative sequences were assigned taxonomic classification at 98% using greengenes reference database of 16S rRNA gene sequences[11]. To remove bias associated with uneven sequencing depth, 128,557 sequences were included in downstream analysis from each sample. The weighted and unweighted UniFrac distance/dissimilarity matrices[12] were constructed after comparing taxon abundance and their presence or absence respectively across samples in pair-wise fashion. Two-dimensional PCoA ordination plots were created to summarize the inter-sample relationships. The Adonis test was used for finding significant whole microbiota differences among discrete categorical (genotype) or continuous variables (gene expression data).

References used for supplementary material

1. Schwiertz A, Taras D, Schafer K, Beijer S, Bos NA, Donus C *et al.* Microbiota and SCFA in lean and overweight healthy subjects. *Obesity (Silver Spring)* 2010; **18**(1)**:** 190-195.

2. Holmes JL, Van Itallie CM, Rasmussen JE, Anderson JM. Claudin profiling in the mouse during postnatal intestinal development and along the gastrointestinal tract reveals complex expression patterns. *Gene expression patterns : GEP* 2006; **6**(6)**:** 581-588.

3. Pan PW, Rodriguez A, Parkkila S. A systematic quantification of carbonic anhydrase transcripts in the mouse digestive system. *BMC Mol Biol* 2007; **8:** 22.

4. Than BL, Goos JA, Sarver AL, O'Sullivan MG, Rod A, Starr TK *et al.* The role of KCNQ1 in mouse and human gastrointestinal cancers. *Oncogene* 2014; **33**(29)**:** 3861-3868.

5. Hansen JJ, Holt L, Sartor RB. Gene expression patterns in experimental colitis in IL-10-deficient mice. *Inflammatory bowel diseases* 2009; **15**(6)**:** 890-899.

6. Hashimoto T, Perlot T, Rehman A, Trichereau J, Ishiguro H, Paolino M *et al.* ACE2 links amino acid malnutrition to microbial ecology and intestinal inflammation. *Nature* 2012; **487**(7408)**:** 477-481.

7. Salcedo R, Worschech A, Cardone M, Jones Y, Gyulai Z, Dai RM *et al.* MyD88-mediated signaling prevents development of adenocarcinomas of the colon: role of interleukin 18. *The Journal of experimental medicine* 2010; **207**(8)**:** 1625-1636.

8. Shroyer NF, Wallis D, Venken KJ, Bellen HJ, Zoghbi HY. Gfi1 functions downstream of Math1 to control intestinal secretory cell subtype allocation and differentiation. *Genes & development* 2005; **19**(20)**:** 2412-2417.

9. Fukata M, Shang L, Santaolalla R, Sotolongo J, Pastorini C, Espana C *et al.* Constitutive activation of epithelial TLR4 augments inflammatory responses to mucosal injury and drives colitis-associated tumorigenesis. *Inflammatory bowel diseases* 2011; **17**(7)**:** 1464-1473.

10. Caporaso JG, Kuczynski J, Stombaugh J, Bittinger K, Bushman FD, Costello EK *et al.* QIIME allows analysis of high-throughput community sequencing data. *Nature methods* 2010; **7**(5)**:** 335-336.

11. McDonald D, Price MN, Goodrich J, Nawrocki EP, DeSantis TZ, Probst A *et al.* An improved Greengenes taxonomy with explicit ranks for ecological and evolutionary analyses of bacteria and archaea. *The ISME journal* 2012; **6**(3)**:** 610-618.

12. Lozupone C, Knight R. UniFrac: a new phylogenetic method for comparing microbial communities. *Applied and environmental microbiology* 2005; **71**(12)**:** 8228-8235.

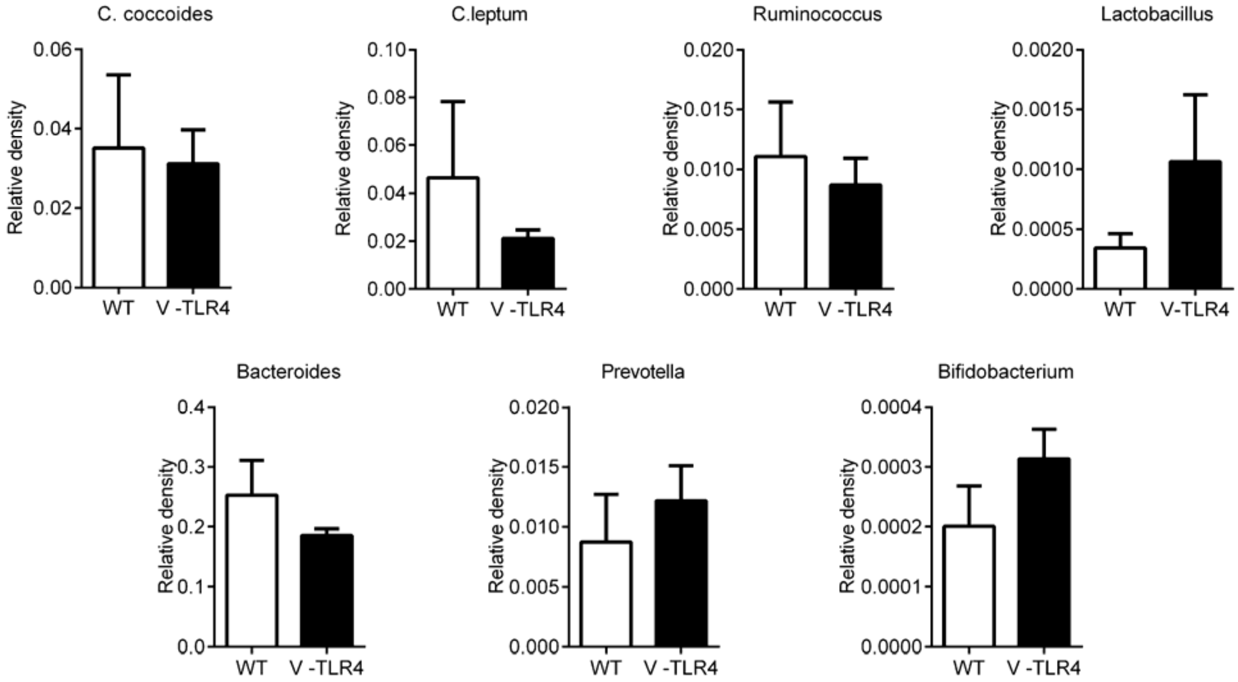

Figure S1: Quantification of bacterial groups in the distal lumen of villin-TLR4 and WT littermates. Relative density of each bacterial group was calculated by dividing the 16S copy number of bacterial group per mg of lumen content with 16S copy number of total bacterial load per mg of lumen content.

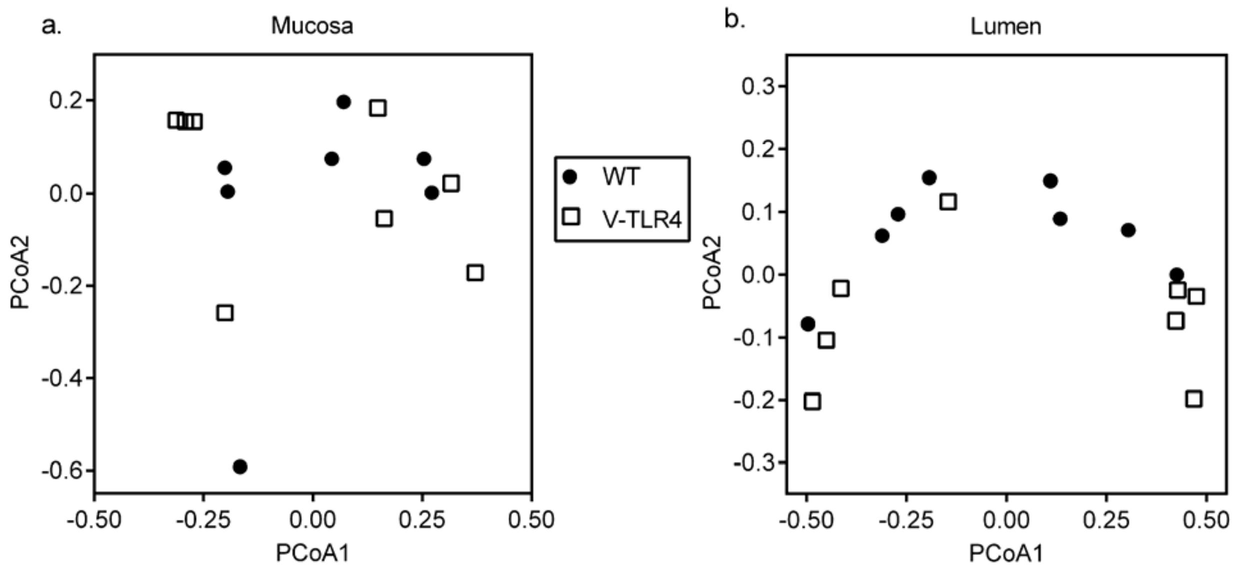

Figure S2: Relative positioning of villin-TLR4 and WT littermate mice in two-dimensions. Principal coordinate analysis (PCoA) based on weighted UniFrac distances (relative abundance of difference bacterial OTUs) between OTUs detected from mucosa (a) and lumen (b) of villin-TLR4 and WT littermates was used to generate ordination plots.

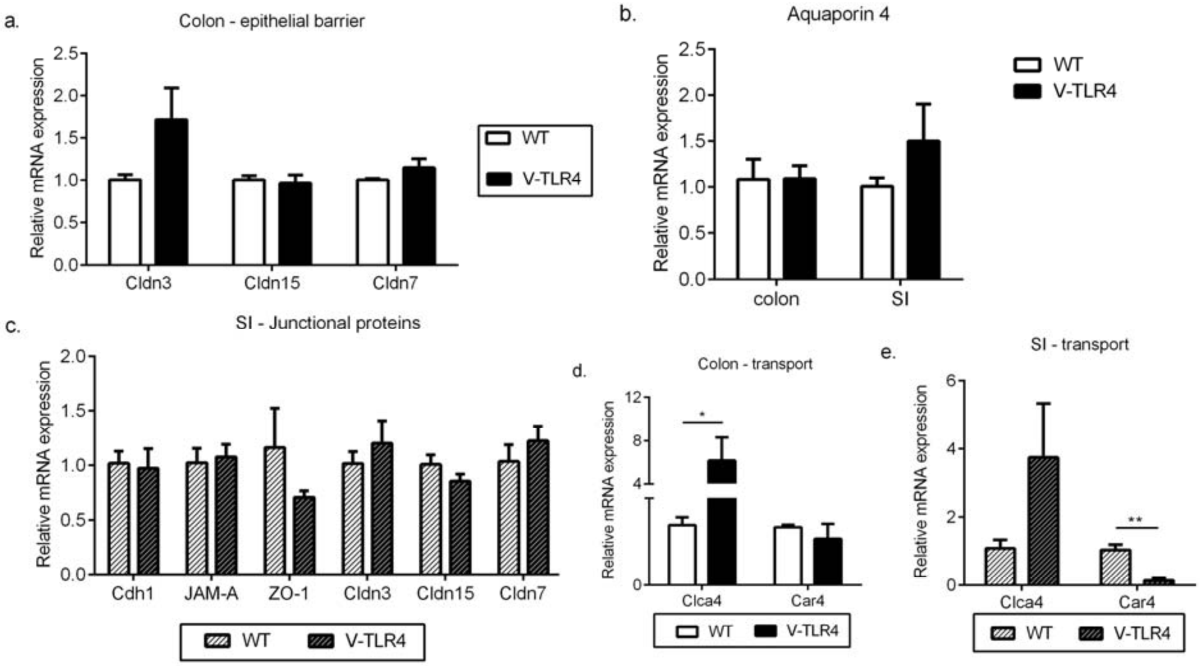

Figure S3: Epithelial gene expression in villin-TLR4 and WT littermate mice. Relative mRNA expression levels of (a) claudin genes in the colonic epithelial cells (ECs); (b) aquaporin 4 ion-transporter gene in both colonic and small intestinal ECs; (c) epithelial junctional proteins in the small intestinal ECs; (d-e) Clca4 and Car4 in colonic and small intestines ECs of villin-TLR4 and WT mice respectively. Results represent mean ± s.e.m of relative mRNA expression normalized to Gapdh and β-actin (n=4-5 mice per group).

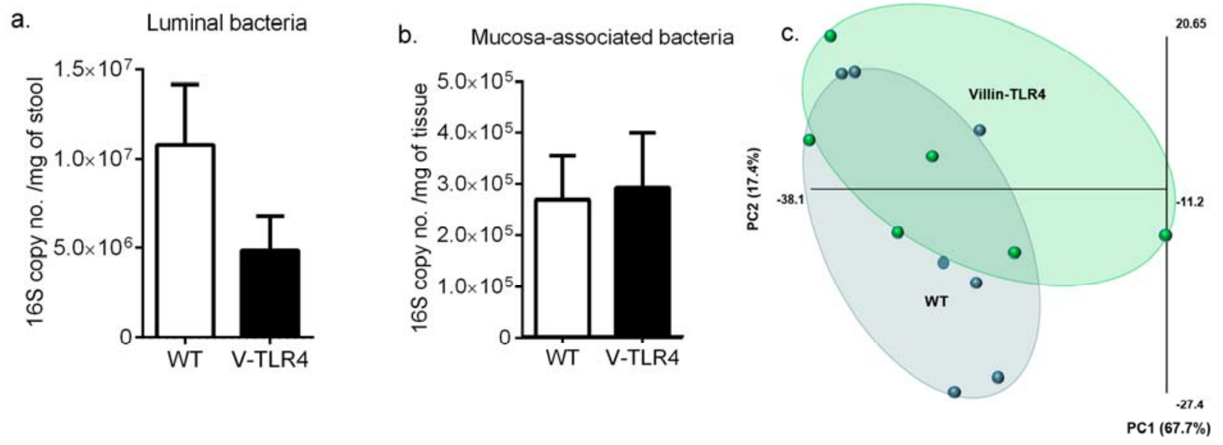

Figure S4: Bacterial composition in the ileum region of villin-TLR4 and WT littermate mice. Bacterial load in the (a) lumen and (b) mucosa of ileum of villin-TLR4 and WT littermate mice. (c) PCA plot generated from T-RF profiles obtained by T-RFLP analysis of luminal microbiota of the ileum of villin-TLR4 (green) and WT (blue) littermate mice.

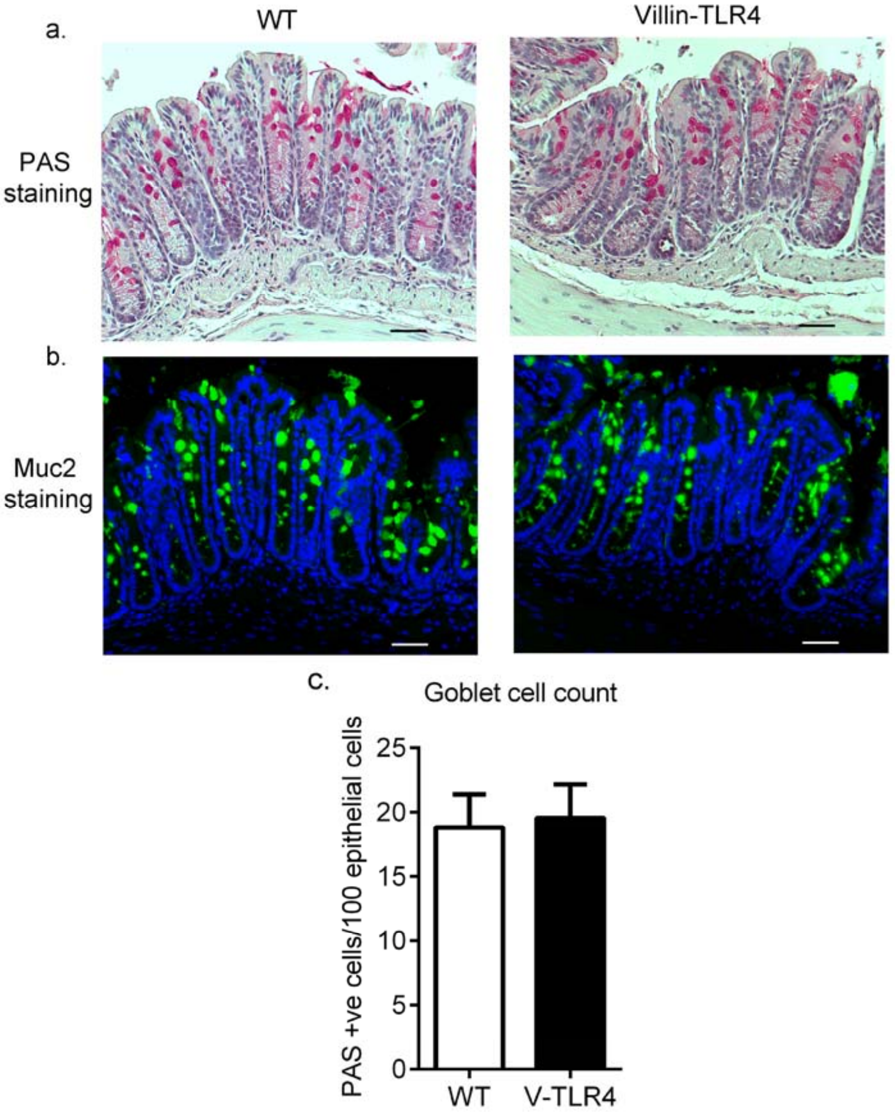

Figure S5: Goblet cell differentiation in villin-TLR4 and WT littermates. Goblet cells in the distal colon of villin-TLR4 and WT mice as revealed by (a) Periodic-acid Schiff (PAS) and (b) Muc2 (green) immunofluorescence staining. (c) Graph of goblet cell number (mean ± s.e.m) counted from PAS positive cells in the distal colon section of the WT and villin-TLR4 mice. For image (b) nuclear cells of intestine were stained with DAPI (blue). Images are representative of four mice per group. Scale bar: 200 μm. Magnification: 20X.

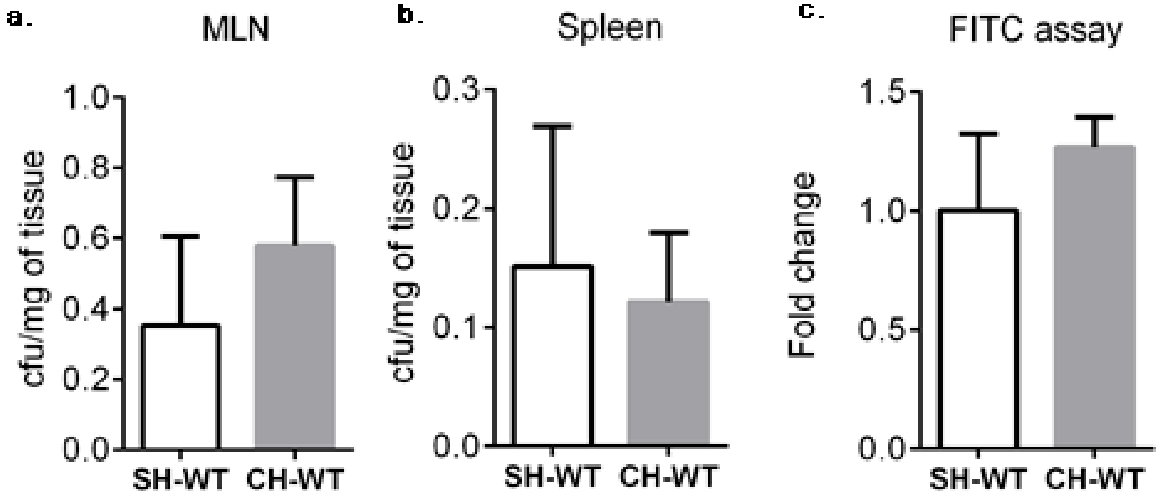

Figure S6: Bacterial translocation and intestinal permeability in cohoused (CH-WT) and separately housed wild type (SH-WT) mice. Bacterial translocation as revealed by Bacterial colony forming units (CFU) formed after aerobic and anaerobic culture of (a) MLNs and (b) spleen. Data represents average number of cfus (mean ± s.e.m) formed on aerobic and anaerobic plates per mg of sample (c) Intestinal mucosal permeability was measured by 4KDa FITC-dextran levels in the serum of SH-WT and CH-WT mice. Results represent mean ± SEM of fold change in FITC-dextran levels relative to SH-WT. N=5-7 mice per group.
